# Jan van der Noordaa (1934–2015); A Virologist *Pur* *Sang*

**DOI:** 10.3390/v7092859

**Published:** 2015-09-15

**Authors:** Ben Berkhout, Michael Bukrinsky

**Affiliations:** 1 Laboratory of Experimental Virology, Department of Medical Microbiology, Academic Medical Center, University of Amsterdam, 1105 Amsterdam, The Nederlands; b.berkhout@amc.uva.nl; 2 Department of Microbiology, Immunology and Tropical Medicine, GWU School of Medicine and Health Sciences, Washington, DC 20037, USA; mbukrins@gwu.edu

**Figure:**
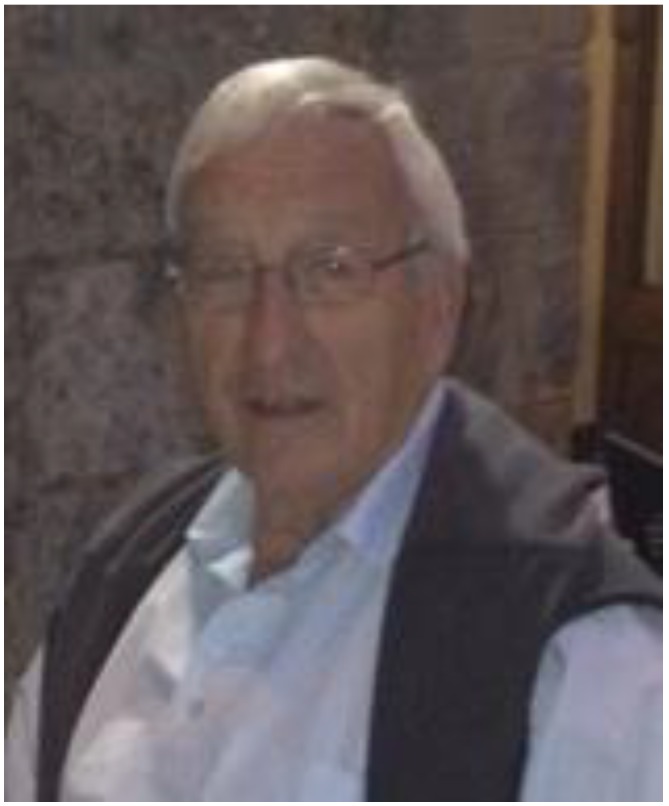
Jan at“Il Palio” (Sienna, Italy, September 2013).

Our loyal friend and colleague, Jan van der Noordaa, passed away unexpectedly at the age of 80 on the evening of 17 June 2015. Jan died in his sleep while visiting his long-term colleague and dear friend, Dr. Alla Bukrinskaya, in Potomac (MD, USA). Jan was born in Leiden (the Netherlands) in 1934, studied medicine at Leiden University and received his MD in 1960. The PhD thesis entitled “Primary vaccination of adults with an attenuated strain of vaccinia virus” was defended in 1964 at the University of Amsterdam. The research was performed as part of his military service and took place under the supervision of Professor F. (Flap) Dekking at the Laboratory of Public Health (Gezondheidsleer) at the University of Amsterdam. His promotor was Professor A. Ch. (Charlotte) Ruys, one of the first female professors in medicine in the Netherlands, Director of the Laboratory and a strong advocate for public health and microbiology.

Jan next crossed the ocean to work in the laboratory of Nobel laureate, John Enders, at Harvard Medical School. Enders pioneered techniques for studying viruses in the laboratory and revolutionized virology, paving the path to the development of vaccines against many serious diseases (including the polio and measles virus). Jan studied the herpes simplex virus and the process of cellular transformation by SV40, a polyomavirus from monkeys that became a model system for molecular cancer research. He continued the SV40 studies in collaboration with the late George Khoury, another distinguished virologist. Jan was appointed Professor of Virology at the University of Amsterdam in 1979 and later became Chair of the Department of Medical Microbiology at the Academic Medical Center (AMC). He introduced the techniques of molecular biology to the field of virology, which allowed probing the length of the DNA genome of human cytomegalovirus, and used restriction enzyme analysis to demonstrate virus transmission by renal allografts. These techniques provided him with an opportunity to pursue the molecular mechanisms of viral replication, e.g., describing the regulation of cytomegalovirus and SV40 gene expression. With Jaap Goudsmit, Jan published one of the first HIV-1 studies in 1986, and his first manuscript on human papilloma virus appeared in 1987 with Jan ter Schegget. Jan’s manuscript based on the pioneering work of René Boom deserves special attention. This paper dealt with a rapid and simple method for purification of nucleic acids and was published in the Journal of Clinical Microbiology in 1990. It received over 3300 citations over the years, peaking at 197 hits in 2007 and still some 130 hits in 2014. The Boom method became very popular in both research and diagnostic settings and the underlying patent was licensed to industry. The revenues obtained by the AMC were instrumental for the construction of modern microbiology laboratories, including Biosafety and Molecular Diagnostic facilities that are currently in use by the Department of Medical Microbiology.

However, the first priority for Jan was public health, and he always thought about patients being the ultimate beneficiaries of his virology research. He was an outstanding research administrator, combining scientific wisdom with respect for young scientists, which, together with a good humor made communication with him a true pleasure for everybody. In the early HIV-AIDS days, he lobbied vigorously to raise funding for expansion of the virology laboratory at the AMC. Asked about the uniqueness of the Amsterdam HIV-1 research, he put things in perspective, indicating that the AMC was probably not unique, as there were luckily many HIV-1 research groups worldwide, but that the AMC certainly had very unique patient material. These serum samples were collected as part of a hepatitis B vaccination study among homosexual men in Amsterdam, which later turned out to be the risk group for HIV-1. He was instrumental in setting up a multi-disciplinary research team around the Amsterdam Cohort Studies, and his seniority was frequently called upon to modulate disputes between the young and ambitious immunologists, epidemiologists and virologists.

Jan van der Noordaa won the Beijerinck Virology Prize of the Royal Dutch Academy of Sciences (KNAW) in 1989 and he received a royal decoration (Ridder in de Orde van de Nederlandse Leeuw) upon retirement from the University of Amsterdam in 1995. The retirement speech covered the broad virology palette that he had established, from poliovirus to herpes virus and from papilloma virus to HIV-AIDS. But he also reminded us of one of the largest accomplishments of modern medicine that took place during his career: the smallpox vaccination campaign that led to global eradication of this virus in 1979. On the occasion of his retirement, he advised the University of Amsterdam and its AMC Medical Center to invest in both clinical and molecular virology. Jan remained active on many national advisory boards, e.g., the Health Council of the Netherlands that among other things advised on the implementation of the vaccine against human papilloma virus, the causative agent of cervix carcinoma.

Jan is remembered for his keen interest in virology and the role of virology in public health. When being invited to present a lecture or attend a scientific meeting, he used to say that after retirement his thoughts were far from science. However, he closely followed all the latest developments in virology and was very eager to hear and discuss research news. His mind was sharp and critical until his last day. He will be remembered for his scientific integrity and he could be a critical colleague, but always with personal charm. He mentored and furthered the careers of many fellow scientists.

His beloved wife Henriëtte Jacqueline Kukler died in 2011. Our hearts and condolences are with his four children, grandchildren and beloved ones.


Prof. Ben Berkhout and Prof. Michael Bukrinsky

